# Noise in Cognition: Bug or Feature?

**DOI:** 10.1177/17456916241258951

**Published:** 2025-03-04

**Authors:** Adam N. Sanborn, Jian-Qiao Zhu, Jake Spicer, Pablo León-Villagrá, Lucas Castillo, Johanna K. Falbén, Yun-Xiao Li, Aidan Tee, Nick Chater

**Affiliations:** 1 Department of Psychology, University of Warwick; 2 Department of Computer Science, Princeton University; 3 Cognitive, Linguistic and Psychological Sciences, Brown University; 4 Faculty of Social and Behavioural Sciences, University of Amsterdam; 5 Warwick Business School, University of Warwick

**Keywords:** noise, variability, cognition, perception, sampling process

## Abstract

Noise in behavior is often considered a nuisance: Although the mind aims for the best possible action, it is let down by unreliability in the sensory and response systems. Researchers often represent noise as additive, Gaussian, and independent. Yet a careful look at behavioral noise reveals a rich structure that defies easy explanation. First, in both perceptual and preferential judgments sensory and response noise may potentially play only minor roles, with most noise arising in the cognitive computations. Second, the functional form of the noise is both non-Gaussian and nonindependent, with the distribution of noise being better characterized as heavy-tailed and as having substantial long-range autocorrelations. It is possible that this structure results from brains that are, for some reason, bedeviled by a fundamental design flaw, albeit one with intriguingly distinctive characteristics. Alternatively, noise might not be a bug but a feature. Specifically, we propose that the brain approximates probabilistic inference with a local sampling algorithm, one using randomness to drive its exploration of alternative hypotheses. Reframing cognition in this way explains the rich structure of noise and leads to the surprising conclusion that noise is not a symptom of cognitive malfunction but plays a central role in underpinning human intelligence.

Psychological scientists constantly battle the variability in human behavior. In experimental studies, no matter what the participants’ task is, whether it is perceptual, a choice between monetary gambles, a questionnaire, and so on, the responses that are produced are not constant. This limits the precision with which most psychological effects can be studied: There are only very few interesting effects that can be studied using a small number of trials and a small number of participants—instead, researchers need to average over either large numbers of trials or large numbers of participants (and sometimes both) to reliably study experimental manipulations.

Individual differences, of course, contribute to this variability, as does randomness in the environment, but variability also exists within an individual when repeatedly facing very similar choices—this is what we term “noise.” Asking participants to perform the same task on multiple occasions, even when those occasions are close together in time, produces surprisingly noisy behavior. In perceptual studies, it is perhaps to be expected that asking participants about difficult-to-perceive stimuli produces noisy responses. But substantial noise also occurs in tasks in which perceptual acuity is no barrier. For example, people’s judgments of the probability of the same event vary from one occasion to another, even when no new information has been observed between judgments (Sundh et al., 2023). Even people’s preferences between monetary gambles will show considerable noise over short periods of time (Loomes & Sugden, 1998; Mosteller & Nogee, 1951; Rieskamp et al., 2006).

Many psychological scientists treat this noise as an afterthought. The mind is often considered to have a deterministic core, with the interesting aspects of behavior being changes to mean responses as a result of experimental manipulations or stable individual differences in mean responses. Noise is considered a nuisance variable by researchers, and thus, following common statistical practice for nuisance variables, noise for continuous responses is often characterized as additive, Gaussian, and independent (Howell, 2017). More formally, many models of cognition can be described as producing a response, R , that is a deterministic function, f , of the stimulus, S . A random variable, ϵ , is added to each response, which represents the additive, independent Gaussian noise (with a mean of zero and a certain variance $\sigma^{2}$ ): R=f(S)+ϵ . Decisions between multiple alternatives are often treated analogously, with covert estimates of the value of each alternative corrupted by additive, independent Gaussian noise (e.g., Drugowitsch et al., 2016; Hey & Orme, 1994).

Statistically speaking, this is a reasonable starting point: Gaussian distributions naturally arise when many random variables (with finite variance) are summed together, even if the distribution from which they are all drawn is non-Gaussian. This could, for example, reflect the combination of many errors in the sensory and response systems (e.g., Stengård & van den Berg, 2019). However, as we review below, studies investigating noise at a deeper level have revealed a rich structure. What has been increasingly recognized is that the noise in cognition is often not noise added in either the sensory or response systems but the noise present in the cognitive computations themselves. In addition, it has been shown that noise is often not Gaussian, not independent, and indeed has other interesting structure.

Explaining these deviations is nontrivial, and doing so can, we argue, reveal crucial insights into how the cognitive system works. We discuss how stochastic approximations to Bayesian inference—local sampling algorithms—have recently been used to account for many aspects of cognition and many features of the noise it generates. For example, and as we discuss in more detail below, local sampling algorithms produce sample-by-sample autocorrelations in which the next state is often similar to the current state, which violates independence in a human-like way. More sophisticated local sampling algorithms have mechanisms to occasionally make bigger steps, and as a result, the overall distribution of steps is not Gaussian. The success of these approaches implies that an even stronger change to the common viewpoint is needed: Not only is noise unavoidable and interesting to study, but the presence of noise in cognition may well be essential to cognitive functioning—allowing a local sampling algorithm to explore alternative hypotheses about the world.

## Noise Has a Rich Structure

Although the above simple formulation of the nature of noisy behavior—deterministic cognition corrupted by independent and additive Gaussian noise—is convenient, it is, as we have suggested, often wrong. First, we point out that the most obvious sources of noise, the sensory and response systems, are not always the largest. We then point out that the common formulation of noise itself is incorrect: Noise is often not additive, not Gaussian, and not independent.

### Types of noise

**Fig. 1.**
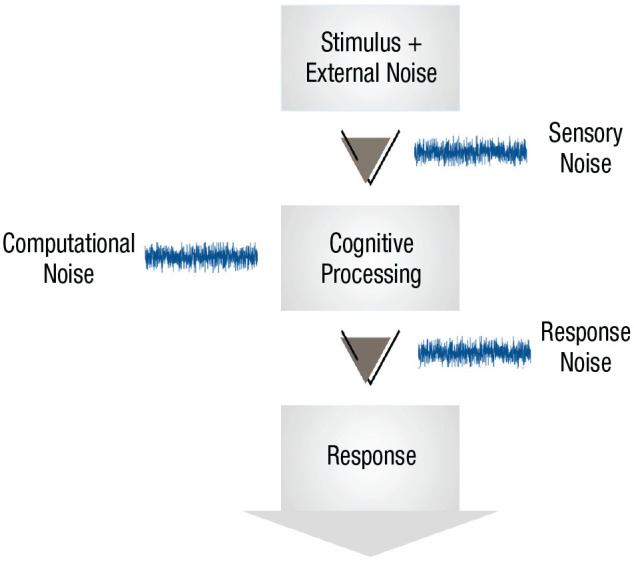
Illustration of information-processing stages and points at which sensory, computational, and response noise are introduced. Unlike external noise, which is added to the stimulus, these three forms of noise are internal to the organism.

We discuss three types of noise: sensory noise, response noise, and computational noise (see Fig. 1). All three types of noise are internal to the individual—that is, they introduce variability in the response to a fixed stimulus. This differs from *external noise*, or noise that is added by the experimenter. There are many kinds of external noise. For example, in investigations of numerosity, participants are very briefly presented with a large number of “dots” on a computer screen and are asked to guess how many dots appeared. People’s responses are often measured as a function of the number of dots that were shown, and confounding aspects of the stimuli such as dot diameter or dot position are often randomly chosen so as not to be reliable cues—however, because participants do use them to some extent this will introduce variability into their judgments (Gebuis & Reynvoet, 2012; Ratcliff et al., 2018). Other kinds of external noise may include the random ordering of trials in the experiment (if participants’ responses are affected by the content of neighboring trials; e.g., Stewart et al., 2002) or even the noise in the number of photons emitted by a visual display. These various kinds of external noise need to be taken into account when assessing the level and type of internal noise.^1^ Separately, researchers need to account for learning—changes to the mean response of the system—which is separate from internal noise but will also cause responses to change between repeated responses to the same stimulus, although in a more predictable way. We keep these potential confounds in mind as we venture now into our discussion of internal noise.

#### Sensory noise

**Fig. 2.**
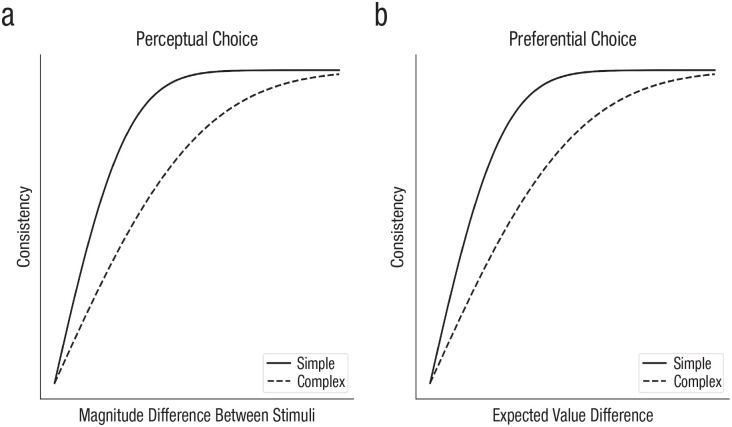
Consistency of choices depending on task complexity in perceptual- and preferential-choice tasks.

Sensory noise, which we define functionally as noise occurring before the important cognitive computations, has been long investigated and identified as a fundamental component of perception (Ashby & Lee, 1993). The key dependent variable in such studies is often the *psychophysical threshold*, or the magnitude of a stimulus property necessary for choices to reach a desired accuracy level. As illustrated by both of the curves in Figure 2a, accuracy in identifying one of two stimuli is generally a smooth function of the difference between stimuli, and the psychophysical threshold is the difference between stimuli that produces a target accuracy/consistency level (e.g., 75% correct). A basic paradigm for investigating sensory noise has thus been detection or discrimination tasks: In visual tasks, participants are asked to report the presence or absence of a very low-contrast stimulus or to choose which of a set of presented stimuli have higher contrast (Pelli & Farell, 1995).^2^

One limiting factor on human performance is the noise introduced by the sensory organs; for example, the number of photons that reach the retina from a light source (e.g., a stimulus presented on a computer screen) follows a Poisson distribution. Initial models supposed that decisions were made optimally and limited only by this external photon noise (de Vries, 1943; Rose, 1948), whereas later *ideal observer* approaches modeled internal inefficiencies in the early visual system, such as information loss resulting from the optics of the eye and noise in photoreceptors. These ideal observer models describe the task faced by the agent and the structure inherent in the task environment and derive optimal solutions, and these normative benchmarks are then compared to empirical data (for variants of this research program, see, e.g., Chase et al., 1998; Chater & Oaksford, 1999; Geisler, 2011). Both of these approaches, however, predicted psychophysical thresholds that were much lower (i.e., meaning better performance) than that shown by experimental participants (Geisler, 2003): Instead, the results pointed to later internal noise, perhaps in the sensory pathways, that affects sensory information (Barlow, 1957).

Although the obvious approach for controlling for external noise is to reduce it to the lowest level possible, a methodological insight for investigating internal noise was to instead add enough external noise to swamp the uncontrollable effects of photon noise and early visual inefficiencies and thereby separate the effect of internal noise from suboptimal calculation. This approach was used in influential models of perceptual decision-making—based on ideal observers—with perceptual templates to convert a stimulus into a single-dimensional signal, followed by additive internal noise and a decision threshold. However, in simple versions, whether the noise was sensory or occurred later was not identifiable (Ahumada & Watson, 1985; Barlow, 1978; Gold et al., 1999).

#### Response noise

Later work in perception, with more complex models, demonstrated that sensory noise alone was insufficient to account for human data. Instead, response noise, acting between the perceptual template and the decision threshold, was deemed necessary to account for experimental data (Lu & Dosher, 1998). Response noise is variability introduced after the important cognitive computations; for continuous responses, response noise can be added to the continuous value of the intended response, whereas for discrete responses it can be captured by the probability of making an unintended response (e.g., general lapses; sometimes termed “tremble noise”). In addition, a second source of response noise can arise by assuming that participants make a continuous covert estimate for each response alternative before choosing a response on the basis of these covert estimates (e.g., estimating the numerosity, or number of dots, in a briefly presented display before deciding whether it is higher or lower than a threshold, or estimating the numerosity of two stimuli separately before choosing the stimulus with the higher numerosity). Response noise can also be the noise added to these covert estimates, and this noise, unlike general lapses, means that a pair of stimuli with more similar covert estimates will have more variable binary responses (see the shape of the curves in Fig. 2; Blavatskyy & Pogrebna, 2010).^3^

Although we have thus far focused on perceptual decision-making, studying the noise in preferential decision-making provides an interesting complement. In preferential decision-making tasks, participants can be given full information about the gamble, and the information needed to make a decision can be presented symbolically (e.g., using Arabic numerals). Therefore, aside from occasional lapses of attention, sensory noise is close to zero for the calculations that are behaviorally relevant. This makes testing for other types of noise easier, although because experimenters ask for participants’ preferences rather than correct answers, there is an additional difficulty in modeling what participants intend to choose.

The classic study of Mosteller and Nogee (1951) established that participants show inconsistent preferences between options even when there is clear sensory information. Here, participants were given the choice of whether to play a game in which they needed to beat a particular hand of poker (played with dice), with fixed gains and losses if they played and no change if they declined. Participants’ choices were not only inconsistent but also less consistent the closer the expected values of the two alternatives were to one another, as illustrated in Figure 2b. A subsequent review of the literature established that when choosing between two alternatives with similar expected values, participants reversed their preferences on approximately 25% of trials when faced with the same alternatives a second time (Rieskamp et al., 2006). Responses have also been found to be more unpredictable when participants are under cognitive load (Olschewski et al., 2018).

Establishing that these inconsistencies are due to noise requires removing the potential confounds of short- or long-term deterministic changes in preference. A careful analysis showed that even when accounting for the long-term effect of participants tending to choose less risky alternatives when making repeated choices, more than 14% of repeated responses remained inconsistent with one another, indicating stochastic choice (Bardsley et al., 2009; Loomes et al., 2002). Other work controlled for both short-term learning on the basis of the identity of the previous trial as well as long-term learning, finding that a substantial amount of inconsistency remained even between the 11th and 12th repetition of choice trials presented in a consistent order (Spicer et al., 2024).

Both types of response noise discussed above have been used to explain these inconsistencies. Constant (or tremble) noise, which results in pressing the wrong key on a fixed percentage of trials regardless of the stimuli due to response noise in the motor system. (This also could be due to lack of attention, misreading, and so on.) Multiplicative noise (termed “Fechnerian noise” in this domain because of the multiplicative noise embodied in the Weber-Fechner law) has been thought to arise from imprecision in calculation. These types of response noise added to a deterministic core, often the normatively motivated subjective expected utility model, have been called “true and error” models (Becker et al., 1963; Birnbaum & Bahra, 2012; Harless & Camerer, 1994).

#### Computational noise

In contrast to sensory and response noise, *computational noise* is noise arising from the cognitive operations that map from sensory input to responses. Drugowitsch et al. (2016) investigated whether noise also occurred in the cognitive computations. The experimental design was based on the classic weather prediction task in which probabilistic cues needed to be combined to determine which response is correct, but with the number of cues and the number of responses both manipulated. Using an ideal observer model of the task, sensory noise was assumed to be Gaussian noise in the perception of the cue (i.e., the orientation of a Gabor patch) that influenced the posterior probability of every response category. Response noise was assumed to be Gaussian noise independently added to the final accumulated posterior probability for each response category. Computational noise was subtly different: It was assumed to be Gaussian noise independently added to the log likelihood of each category separately after each piece of evidence was observed, allowing it to accumulate over the sequence of cues. Multiple cues had higher thresholds than single cues (illustrated in Fig. 2a) and in such a way that extensive model comparison revealed that almost all of the deviations from the ideal Bayesian model could be explained by computational noise.

Because these computational inefficiencies could potentially also be explained by deterministic calculations that were poorly adapted to the task (e.g., Beck et al., 2012), Drugowitsch et al. (2016) also included the key manipulation of presenting the exact same trials to participants on multiple occasions. Supplemental analyses demonstrated that participants did not change how they did the task, and so the analysis was able to decompose the extent to which deviations from the correct Bayesian model (aside from sensory and response noise) were either deterministic or stochastic. Two thirds of the noise in behavior was due to stochasticity in inference—thus, the leading cause of inefficiency was computational noise. Stengård and van den Berg (2019) later generalized this result to visual search, finding a similar magnitude of computational imperfections under unlimited viewing conditions but a higher percentage contribution of sensory noise when viewing time was limited.

Separately, there is evidence for computational noise in addition to response noise in risky choice. An empirical fact that counters the sufficiency of response noise in explaining choice inconsistency is the reliability of choice when one option dominates the other. *Dominance*, specifically first order stochastic dominance, means that one alternative has a higher probability of providing an outcome at least as good as its alternative on any potential comparison value. For example, one alternative with a 15% chance of £30 and a 10% chance of £10 and otherwise nothing would exhibit dominance over an alternative with a 10% chance of £30 and a 15% chance of £10 and otherwise nothing. In these cases, participants are very rarely inconsistent, for example, 2.39% in Loomes and Sugden (1998) and 5.48% in Spicer et al. (2024).

This fact, combined with the greater inconsistency without dominant alternatives, suggests that response noise alone is insufficient to explain variability in preferential choice because the relationship between conditions echoes that found in perceptual choice (illustrated in Fig. 2). Explanations include a more complex process that modulates response noise depending on the option pairs (Blavatskyy, 2011) or the inclusion of a dominance-detection mechanism in the model (Kahneman & Tversky, 1979). A more parsimonious solution, however, is to go beyond adding variability to the utilities and make the function used to convert stated values to utilities itself variable (Becker et al., 1963). For these kinds of random preference models, there is a strict ordering of the utilities of alternatives for a given utility function, but a different utility function is sampled each time a choice is made. If every possible utility function respects dominance, a response based on sampled utility functions will respect dominance, whereas more ambiguous choices will lead to noisy responses (Loomes, 2015), although this assumption can be softened so that a different utility function is sampled at each time step in a decision process (Navarro-Martinez et al., 2018). The random selection of utility functions in these models is a form of computational noise.

Preferential-choice studies have also investigated noise in situations in which participants learn about decision alternatives from experience instead of through description, for example, when presented with two options that are rewarded with different probabilities. Behavior in these *bandit tasks* is known to be noisy, with participants tending not to always select the alternative that provides the highest probability of reward but instead selecting alternatives roughly proportional to their probabilities (Vulkan, 2000). More recent work investigated a bandit task in which participants selected between options with values that smoothly changed over time. Different reinforcement learning models were compared, with the model that matched human behavior the best including multiplicative noise in the updates made to value functions (Findling et al., 2019). Follow-up work showed that multiplicative computational noise played an adaptive role in tasks in which the rewards changed in volatile ways. This type of noise is also computational because the noise values do not transiently affect responses but appear to be incorporated into participants’ representations (Findling et al., 2021).

### Functional form of the noise

Although the above studies have provided evidence for the existence of computational noise, it is difficult to determine its exact characteristics. This is because different noise distributions can produce very similar effects with binary choices (e.g., Luce, 1959). Therefore, for the purposes of investigating the functional form of the noise, such as whether it is Gaussian and independent, it is more revealing to investigate the variability of people’s estimates.

#### Non-Gaussian noise

Probabilities form a component of normative models of perceptual and preferential decision-making and are often directly presented to participants in studies of preferential decision-making. Therefore, the noise in probabilities is of interest to researchers in both perceptual and preferential domains, and a reasonable starting point is to assume that probability estimates are affected by Gaussian noise.

**Fig. 3.**
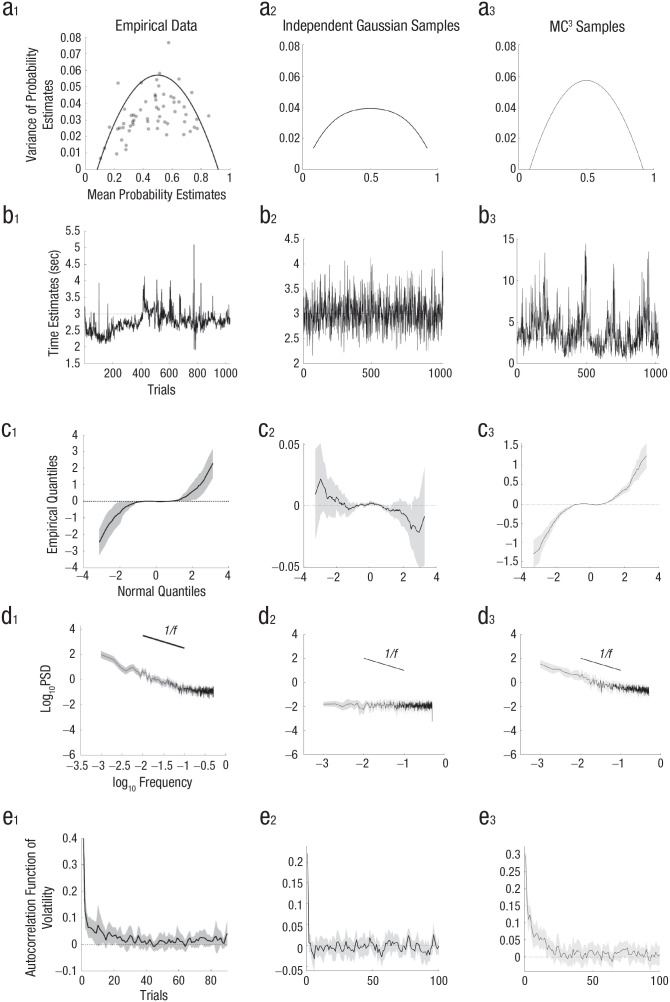
Illustrations of the functional form of the noise in cognition. The mean and variance of repeated probability estimates (a) show an inverted-U relationship (data from Sundh et al., 2023, Experiment 3), whereas Gaussian error (even if restricted to the response range) produces a flatter function at the level of noise observed in participants. MC^3^ samples fed through the probability estimation function of the autocorrelated Bayesian sampler model (see Fig. 4) show an inverted-U relationship because of their binomial noise. There are interesting patterns in how estimates change over time (b) from an example participant making repeated estimates of a fixed time interval (3 s; data from Zhu et al., 2022), independent samples drawn from a Gaussian distribution, and MC^3^ samples drawn from a Gaussian distribution. Estimate data are analyzed in different ways in (c through e), with lines for the mean and shaded regions for the 95% confidence intervals. In plots of the deviation of empirical quantiles from standard Gaussian quantiles plotted against standard Gaussian quantiles (c), a flat line indicates a Gaussian distribution, and a positive slope indicates a heavy-tailed distribution. In plots of spectral density analyses (d), a flat line indicates independent noise, whereas a slope of 1/f on the log-log plot indicates long-range autocorrelations. In plots of autocorrelations in the magnitude of change between successive estimates (e), independent noise shows zero autocorrelation at any lag beyond zero, whereas positive autocorrelations indicate volatility clustering. MC^3^ = Metropolis-coupled Markov chain Monte Carlo.

However, in a task in which participants were repeatedly asked to estimate the relative frequency of geometric objects on a screen, the variability of probability judgments peaked for probability estimates in the center of the range (i.e., close to .5) and was well described by a binomial distribution instead of a Gaussian distribution (Howe & Costello, 2020). Although these results were based on perceptual stimuli for which sensory noise could play a role, a later study found the same results when participants were estimating probabilities from memory (e.g., estimating the probability of rain on a random day in England); moreover, additive Gaussian noise could not explain the inverted-U shape, even if responses were censored at the edges of the response range (see Figs. 3a_1_ and 3a_2_; Sundh et al., 2023).

**Fig. 4.**
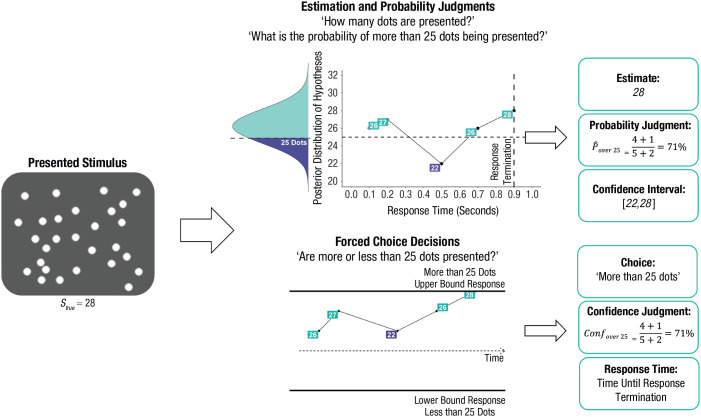
Schematic depiction of the autocorrelated Bayesian sampler (Zhu et al., 2024) and its versatility in accounting for different types of responses, with reference to an example numerosity task. The stimulus (left) is first converted into a psychological distribution (reflected by the Gaussian curve), which can then be sampled to answer various potential queries, including estimates (here, the last sample), decisions (whether the count is above/below 25 based on which is supported by most samples), probability/confidence judgments (the relative proportion of samples for each response plus a prior count of 1 for each alternative that acts as the generic prior), confidence intervals (the empirical quantiles for a given range), and reaction times (based on the total number of samples before reaching the decision boundary).

The appropriateness of additive Gaussian noise has also been questioned in studies of time interval estimation. In these studies, participants are given a short demonstration of a target time interval, such as 3 s, and then asked to reproduce it repeatedly, without feedback, as if they were “drumming” a keyboard key. A plot of successive time interval estimates from a single participant attempting to hit a key every 3 s for 1,000 trials in an experiment from Zhu et al. (2022) is shown in Figure 3b_1_. The distribution of responses looks different from what would occur with Gaussian noise (for comparison, see Fig. 3b_2_).

We can quantify this by looking at the distribution of changes in the time interval estimate between successive responses. If Gaussian noise is added onto participants’ responses, then the difference between successive estimates will also follow a Gaussian distribution.^4^ A nonparametric method to investigate this distribution of changes in successive estimates is to investigate the quantiles of the standardized distribution (i.e., subtracting the mean from each estimate and then dividing each by the standard deviation, ordering the estimates from smallest to largest, and then finding the value that separates the 1st percentile of estimates from the rest, the value that separates the 2nd percentile of estimates from the rest, etc.). These empirical quantiles can be compared to the quantiles generated from the normal distribution (Wilk & Gnanadesikan, 1968), such as the standard normal distribution with a mean of zero and standard deviation of one. If the successive estimates showed Gaussian noise, then subtracting the standard normal quantiles from the empirical quantiles would, on average, produce zeros for each difference in quantiles, and they would not deviate significantly from zero (as in Fig. 3c_2_). However, Figure 3c_1_ shows curved deviations with a positive slope because the empirical quantiles are further from their mean than predicted by a Gaussian distribution (e.g., the standard normal quantile that is three standard deviations below the mean at −3 is actually two more standard deviations below the mean in the empirical distribution, and likewise the standard normal quantile that is three standard deviations above the mean is an additional two standard deviations above the mean in the empirical data). Thus, the distribution of successive changes in time interval estimates has *heavy tails*: Most changes are small, but some changes are very large—more than can be accounted for by a Gaussian distribution (Zhu et al., 2022).

The heavy-tailed nature of changes in successive estimates also occurs when participants are given a new time estimation target on each trial, which changes slowly according to a Gaussian random walk (on log time; Zhu et al., 2021). Heavy-tailed changes also occur in price prediction tasks in which the feedback follows a Gaussian random walk (on log price): Participants’ successive price estimates show heavy-tailed changes (Zhu et al., 2021).

#### Nonindependent noise

Perhaps an even more fundamental assumption than that of Gaussian errors is that the noise in one trial does not depend on the noise in previous trials. However, it has been found that noise does, in fact, depend on previous trials, and not just on the immediately preceding trial. Gilden et al. (1995) showed this in a temporal estimation task with a fixed target using the diagnostic tool of plotting the power spectral density of each frequency on a log-log plot. In this analysis, a Fourier transform is used to decompose the time series of estimates into a weighted sum of a set of oscillating functions, each operating at a different frequency. The weight calculated for each function is the spectral power at that frequency, with power at lower frequencies indicating dependencies at longer times scales (for further details of this analysis, see Gilden, 2001; Wagenmakers et al., 2004). Independent noise will show a flat line in this plot because it has equal power at all frequencies, as shown for independent Gaussian noise in Figure 3d_2_. However, as shown in Figure 3d_1_, participants instead show a slope close to −1 , also termed “ 1/f noise,” which indicates long-range autocorrelations.^5^ Later work showed that long-range autocorrelations and heavy-tailed changes co-occur in both a temporal estimation task as well as a semantic association task (Zhu et al., 2022). Beyond temporal estimation, long-range autocorrelations have been found in the response times of a variety of tasks, and surprisingly, the autocorrelations can explain more of the variance in response time than the experimental manipulations do (Gilden, 1997).

Another demonstration of the nonindependence of noise can be found by inspecting the autocorrelations in changes between successive estimates. Interestingly, although there are almost no autocorrelations in the changes between estimates,^6^ there are autocorrelations in the *magnitude* of the changes between estimates. Although the direction of change of an estimate is hard to predict, large changes (in either direction) are more likely to be followed by large changes, and small changes by small changes. This effect, known as *volatility clustering* (this terminology is drawn from finance; Cont, 2001), occurs in estimates of fixed time intervals (see Fig. 3e_1_), despite this effect not occurring for Gaussian noise (see Fig. 3e_2_). It also occurs for participants’ estimates in tasks in which the (log) target time changes from trial to trial as a Gaussian random walk and in individual price prediction time series when participants predict a Gaussian random walk (on log price; Zhu et al., 2021), despite Gaussian random walks also not exhibiting volatility clustering.

Finally, additional nonindependence can be found in tasks in which participants are asked to generate random sequences. For example, they are asked to randomly generate a sequence using the numbers from one to 10, as if drawing numbers from a hat (with replacement). In this task, people deviate from independence by showing too few repetitions and too many transitions between adjacent numbers, and they too often continue along the same direction on the number line (Wagenaar, 1972). Although many of the results in this section so far have been established only for estimates and not for binary choice, the lack of repetitions in random generation is one that has also been observed with binary outcomes (Rapoport & Budescu, 1997).

## Toward a Unifying Explanation of Computational Noise

From the above discussion, sensory and response noise, either individually or combined, are insufficient to explain the noise in human behavior, and therefore a substantial proportion of the noise has to occur in the cognitive computations instead. We also have seen that noise does not have a simple form and instead is non-Gaussian and shows intricate dependencies. What could account for such complex-seeming noise across such a broad range of tasks?

One approach is to assume that random errors are made in each cognitive calculation, and these errors propagate through subsequent calculations. This approach characterizes models of computational noise both in perceptual and preferential decision-making (Drugowitsch et al., 2016; Findling et al., 2019, 2021). However, so far, there is no unified approach: In perceptual decision-making, an ideal observer model was used, and the noise was applied independently to probabilities, whereas in preferential decision-making, a reinforcement learning model was used, and the noise was applied independently to the inferred values of options. A challenge for this approach of directly adding noise to specific cognitive computations is also to explain why the functional form of the noise in estimates is non-Gaussian and nonindependent in the specific way that has been observed.

A second approach is to assume that participants are randomly selecting different rules or heuristics to apply on each trial (Becker et al., 1963; Rieskamp & Otto, 2006). This approach can explain why variability can be much larger for some kinds of choices (e.g., dominance in preferential choice) than others, but, like directly adding noise to computations, it also would need to explain why the noise in estimates has specific non-Gaussian and nonindependent characteristics.

A third approach is to use sequential sampling models, such as drift-diffusion models. These models are based on the sequential probability ratio test but relax the assumption that all noise is sensory noise. Sequential sampling models have shown much success in fitting data in both perceptual and preferential decision-making (Busemeyer & Townsend, 1993; Navarro-Martinez et al., 2018; Ratcliff & McKoon, 2008). These models have also been augmented with computational noise to explain long-range autocorrelations in response times (Wagenmakers et al., 2004) and can be modified to also produce estimates (Kvam et al., 2022). Although promising, the sequential sampling models that fit these different tasks are hard to reconcile because they differ in what they accumulate (e.g., evidence or utility) and do not yet offer an account of why the noise in estimates is non-Gaussian and nonindependent.

Each of these existing frameworks has had success in explaining specific results based on noise in cognitive computations, but is a more general explanation of noise possible? One principled route to producing such a general explanation is to start with probabilistic models about the world, such as Bayesian or ideal observer models,^7^ but to acknowledge that the brain cannot possibly apply such probabilistic models using exact symbolic calculations using the mathematics of probability theory because these would be computationally intractable. This means an approximation is needed, and one of the most widespread approaches to approximation in computational statistics and machine learning is sampling. It is often possible to successfully draw samples from a probability distribution even when it is far too complex to represent the full distribution (e.g., keeping in mind the probabilities of every possible interpretation of an image). Sampling typically involves using some variant of so-called Markov chain Monte Carlo (MCMC) sampling, a sampling algorithm that starts with a particular hypothesis (e.g., this image shows a cheetah) and makes stochastic and local changes to the hypothesis (e.g., this image shows a leopard). When implemented correctly, this algorithm generates samples from the probability distribution over possible hypotheses while remaining psychologically more plausible than representing the entire probability distribution (Griffiths et al., 2012; Zhu et al., 2023).

This viewpoint postulates that the brain will roughly follow Bayesian probability theory but will be subject to systematic biases resulting from computational constraints on cognition, limiting the number of samples drawn. These biases will arise in a variety of ways: First, small samples will depend on their starting point (because the choice of starting point will only “wash out” after a large sequence of samples has been drawn, so that the entire probability space has been explored); this dependence on starting point has been argued to account for effects such as anchoring and adjustment, sub- and superadditivity, and causal reasoning errors (Dasgupta et al., 2017; Davis & Rehder, 2020; Lieder et al., 2018; Sanborn & Chater, 2016; Spicer et al., 2022; Zhu et al., 2024). Moreover, the brain needs to make appropriate inferences in light of small samples (e.g., not simply assuming that an event that happens, say, twice in a sample of two must occur with certainty). Appropriate correction for small samples leads to regression of probability values from extreme values, which can provide an explanation for the conjunction fallacy, among other effects (Zhu et al., 2020, 2024). Moreover, given small samples, the brain has to extrapolate the probability of new items on the basis of their similarity to the small number of items that have been sampled (in computational statistics, a method known as approximate Bayesian computation), which provides an elegant explanation of the close relationship between judgments of probability and similarity that underlies the representativeness heuristic (Kahneman & Tversky, 1972). More broadly, the idea that human probabilistic reasoning uses a sampling approximation provides an effective explanation of many classic effects in the experimental literature on probability judgments (Chater et al., 2020).

In many ways, the idea of statistical sampling is a generalization of the ideas proposed to explain computational noise. Random errors in a calculation that are subsequently incorporated into later calculations are hallmarks of multistage sampling processes, such as ancestral sampling or sequential Monte Carlo (Daw & Courville, 2008; Prat-Carrabin et al., 2021; Sanborn et al., 2010). Randomly selecting a rule on each trial is also a form of sampling. Additionally, random walk models can be formulated from sampling hypotheses instead of data, providing a link between these approaches and drift-diffusion models (Hamrick et al., 2015; Vul et al., 2014; Zhu et al., 2024). Indeed, regarding the observations about cognitive noise we reviewed above, Findling and Wyart (2021) commented that sampling was a potential contributor to the computational noise found in their task. Stengård and van den Berg (2019) found that simple sampling on its own was the third best model in their model competition for explaining computational noise in perception.

However, although the idea of sampling seems generally well suited to explaining the noise in cognitive computations, to make further headway, the idea of sampling needs to be fleshed out by identifying specific sampling algorithms and quantifying how the outputs of these algorithms map onto behavior. Fortunately, there has been progress made toward both of these goals, particularly when it comes to explaining the functional form of the noise.

### Sampling algorithms with human-like noise

MCMC is a family of algorithms that allows statisticians and researchers to generate samples from an arbitrary target probability distribution. Importantly, MCMC can produce samples from high-dimensional and complex target distributions as long as local information about the likelihood of data points is available. Given its flexibility, MCMC is used widely in statistical inference and indeed has been acknowledged as one of the most important algorithms of the 20th century (Cipra, 2000). Intuitively, MCMC proceeds by iteratively creating a sequence, or chain, of states, in which each state is a sample of the target probability distribution. In each iteration, a proposed state is generated by randomly modifying the last state. The previous state and the newly proposed state are then compared by calculating the ratio of the likelihood of the proposed state given the target distribution to the likelihood of the previous state. In the algorithm’s most common form, Metropolis-Hastings, this ratio is used to stochastically decide whether the proposed state is used as the next state in the sequence of MCMC states or if the last state is retained. Whenever the proposed state is more likely given the target distribution, the proposal is accepted. However, if the proposed state is not more likely, the proposed state is accepted with a probability proportional to the ratio of likelihoods of the proposed and the previous state (Hastings, 1970; Metropolis et al., 1953). Therefore, MCMC will not simply seek the mode of the target distribution using random search but will navigate the target distribution proportionally to its likelihood—usually moving closer toward the target mode but also exploring areas of lower likelihood.

It can be shown that, given mild assumptions about the way that proposals are produced, after many iterations, the generated states correspond to samples from the target distribution. Thus, these samples can be used to estimate properties of the target distribution, such as its mean or variance. However, it is important to stress that the sequence of MCMC states only perfectly matches the target distribution in the limit of an infinite number of samples. For example, because the MCMC procedure needs an arbitrary starting point, the first states in the MCMC sample do not correspond to the target distribution and are commonly discarded. Finally, the samples produced by MCMC are not independent because each sample is produced given its predecessor. As a result, the number of effective samples of an MCMC chain (the number of independent samples that the chain contains) can often be considerably lower than the number of MCMC iterations (Kass et al., 1998).

The standard Metropolis-Hastings MCMC algorithm produces nonindependent samples with the changes between samples not necessarily corresponding to the distribution from which it is drawn. However, an elaboration of this algorithm, Metropolis-coupled MCMC (MC^3^; Geyer, 1991), was developed for the purpose of searching probability distributions with many modes rather than a single peak. This algorithm works by running multiple MCMC chains simultaneously, with one chain drawing samples from the correct target distribution and the remaining chains drawing samples from a “melted” version of the distribution in which it is easier to move between modes of a probability distribution that are separated by regions of very low probability. The remaining chains are useful for exploring the distribution because, when appropriate, one of these remaining chains can swap positions with the MCMC chain sampling from the correct target distribution. In this way, MC^3^ can make occasional large jumps across the hypothesis space, allowing it to also produce heavy-tailed changes even when sampling from a fixed Gaussian distribution (see Figs. 3b_3_ and 3c_3_; however, sampling from a heavy-tailed distribution may be necessary to produce tails as heavy as those observed in Figs. 3b_1_ and 3c_1_; Zhu et al., 2022), or when the target follows a Gaussian random walk in either time interval estimation tasks or price prediction tasks (Spicer et al., press; Zhu et al., 2021).

The multiple chains of MC^3^ introduce human-like long-range autocorrelations (see Fig. 3d_3_), and surprisingly, in both fixed target and random walk target tasks, this local sampling algorithm also produces almost no autocorrelations in the changes between estimates but does show autocorrelations in the magnitude of these changes (see Fig. 3e_3_; Zhu et al., 2021). MC^3^ better fit the overwhelming majority of participants in a price prediction task than nonsampling models of human behavior (Spicer et al., in press). In this analysis, response noise was also included, although it played a minor role, and generally it is important to consider the possibility of all three types of noise when fitting human behavior.

Castillo et al. (2024) showed that “volitional” random generation could be explained as resulting from a related MCMC algorithm. Participants were asked to say the heights of people at random, and several measures of serial independence were calculated from their sequences. For example, Castillo et al. replicated past results that showed that, when compared to true randomness, there are very small distances between successive items in the sequences that people generate, and with long runs following the same ascending or descending direction (Towse & Neil, 1998). The authors compared these deviations to those produced by several MCMC algorithms and found that algorithms running multiple chains with autocorrelated proposals explained people’s sequences best, and better than an existing random-generation model. Furthermore, people’s responses reflected the true distribution of heights, as MCMC algorithms do. This result was bolstered by León-Villagrá et al. (2022), who found that when asked to generate random samples from environmental distributions, people’s sequences reflected both coarse and fine-grained statistics of the environment (e.g., of National Football League scores).

### Mapping samples onto other behavior

The above comparison of MC^3^ to the functional form of noise in participants’ estimates uses a simple mapping from the sampling algorithm to behavior: Each successive estimate is a new sample generated by the algorithm. Furthermore, we have recently proposed that a wide range of empirical findings ranging from psychophysics to high-level cognition can be captured by a cognitive modeling framework that we call the “autocorrelated Bayesian sampler” (see Fig. 4; Zhu et al., 2024) that captures the above judgments, response times, confidence, and many other aspects of behavior within a single framework. According to this framework, the mind first samples hypotheses using an MCMC algorithm such as MC^3^, with the number of samples determining response times. For example, when briefly presented with a display showing many dots on a screen, samples of the number of dots will be drawn using the algorithm. With the drawn samples, the mind then integrates these hypotheses to make responses, with different types of responses involving different types of integration. For example, it might calculate the relative proportion of samples that belong to a given category to produce a probability judgment, average the sample values to produce a point estimate, or compute quantiles to produce confidence intervals.

Although these responses could be made with the drawn samples alone, doing so would occasionally lead to extreme responses resulting from high sampling error with a small number of samples, so a prior on responses is incorporated instead. In intuitive terms, this prior can be seen as a count of assumed samples held before any actual samples are drawn, and it acts as a useful bias: Although it causes a form of conservatism and results in conjunction fallacies, it robustly improves the accuracy of probability judgments overall (Zhu et al., 2020, Appendix C). For example, if judging the probability of rain, the estimate is the number of rainy days sampled plus the prior count of rainy days divided by the total number of samples and prior counts for all responses (see Fig. 4). If the counts are adjusted for the autocorrelation in the samples, then the counts will follow a binomial distribution that produces an inverse-U-shaped relationship between mean probability judgments of an event and the variance of the judgments of that event, as shown in the human data (see Fig. 3a_3_). Furthermore, the observations that there are very few extreme responses and the mean-variance curve falls to zero before reaching the edge of the range can be explained by a prior over responses: knowledge that probabilities tend not to always be zero or one (Kolvoort et al., 2023; Sundh et al., 2023).

This unifying perspective can thus explain the functional form of the noise observed in behavior, and this approach has been extended to binary perceptual choice. In perceptual choice, samples of hypotheses are accumulated until there is enough evidence for one response over the other, borrowing this formulation from sequential sampling models. The autocorrelations of the local sampling algorithm introduce interesting effects, particularly that errors will be, on average, slower than correct responses in specific conditions, as found in human data (Ratcliff & Rouder, 1998). However, this approach has not been used in the tasks used to demonstrate computational noise in perceptual judgment and will need to be combined with the right ideal observer models to evaluate how well they account for noise, and behavior more broadly, in those tasks. Further, this framework will need to be extended to preferential decision-making, although the success of sequential sampling models in this domain, including demonstrations of stable individual differences in parameters across perceptual and preferential choice, suggests that a unification is possible (Busemeyer & Townsend, 1993; Frydman & Nave, 2017; Navarro-Martinez et al., 2018). The hope for the sampling approach is that it not only can explain empirical effects but also could be used to characterize an individual in one task and make accurate predictions about their behavior in very different tasks.

## Is Noise in Cognition a Bug or a Feature?

We have argued that computational noise is a major contributor to the substantial variability in human behavior. It is natural to see noise as the enemy of successful computation and something to be minimized as far as possible (e.g., Kahneman et al., 2022). In everyday life, it limits the ability of an individual to perceive a stimulus correctly, and noise in responding means that an individual’s intended action cannot be perfectly executed. It is clear that when in situations such as needing to identify oncoming traffic and press the correct pedal while driving or perceiving the location of each stair and reliably placing your foot on it, too much noise is harmful. Reducing noise beyond what others do can also be incredibly rewarding: It is part of what separates the highest paid sports professionals, surgeons, or craftspeople from their peers.

From this point of view, the prevalence of computational noise in the brain is viewed as an unavoidable side effect of the noisy nature of individual neurons, or some other aspect of the hardware of the brain (Faisal et al., 2008; Rolls & Deco, 2010). This is the point of view even of optimal Bayesian models of perception and memory that assume that the mind understands the noise that it faces. These models construct the best possible response from noisy input (Ernst & Banks, 2002; Shiffrin & Steyvers, 1997) but will perform better when noise is lower. As an alternative, efficient coding approaches also attempt to mitigate the detrimental effects of noise by encoding information in such a way as to be minimally affected by the noise (Sims, 2016). These two approaches can even be combined so that encoding is efficient and decoding is optimal (Wei & Stocker, 2015), but again, additional noise will negatively affect the resulting behavior.

However, the view of noise has not always been negative; indeed, some researchers studying noise have identified situations in which it could be useful. The most obvious case is in competitive contexts in which being unpredictable is important (e.g., selecting where to kick a soccer ball in a penalty shootout), but the noise can also be useful in the individual tasks we discuss here. For example, Faisal et al. (2008) and others have pointed out that noise is useful in stochastic resonance theory, which assumes that the mind contains a detector that responds only when signal strengths reach a certain threshold: For intermediate signal strengths, a moderate level of noise enhances the chance that they reach a threshold without swamping the signal. However, in this account, noise is not useful above this threshold like it is in the sampling approaches we advocate here. Likewise, Findling et al. (2021) argued that computational noise that scales with value is useful, although perhaps only for making predictions about value in changing environments, and there are other situations in which randomness could be useful (Icard, 2021).

But we argue that noise may better be viewed as a feature rather than a bug, even outside of the restricted scenarios discussed above. Our specific proposal is that the brain manages the probabilistic inference required to deal with a highly uncertain world through sampling—and this process of sampling is, by its very nature, noisy. Indeed, when we view the brain as a Bayesian sampler, the noisiness of human thought and behavior is not a failure to be ironed out but central to the basic operation of human cognition. This argument expands on the point made by Renart and Machens (2014) that if the brain approximates probabilistic inference through sampling, then noise in the brain is a feature rather than a bug—it allows the brain to consider only one or a small number of hypotheses at a time and with probabilities proportional to the relative time spent considering each hypothesis.^8^ This is more psychologically plausible than implementing exact Bayesian inference and is congruent with psychological ideas about considering a single hypothesis at a time (e.g., the singularity principle; Evans, 2007).

The autocorrelations in human behavior are linked to an additional and very plausible constraint—as in theories of mental foraging (Hills et al., 2012), the brain will very often make only small changes to the hypothesis currently under consideration. In this conception, finding the most probable hypothesis is like searching through the hypothesis space, akin to how someone might try to find the highest point in a landscape when it is dark. A natural starting point is “hill climbing”: always moving to the most likely hypothesis you can currently reach, which will eventually result in reaching a “peak” of the probability distribution. However, there is no guarantee that the probability distribution we wish to explore has a single peak—hypotheses likely can cluster together, so mental representations can have multiple peaks. An example is multistable perception, in which there are multiple likely interpretations of a visual stimulus, but a compromise between the two is very unlikely (Gershman et al., 2012). For this kind of landscape, stochastic search may be more efficient than hill climbing because it offers the chance to find new peaks (Y.-A. Ma et al., 2019). This is congruent with the argument that noise could allow the cognitive system to move away from idiosyncratic biases or allow more transitions between strong attractor states in a neural system (Findling & Wyart, 2021). Our demonstration of how sampling algorithms developed to deal with probability distributions with multiple peaks (e.g., MC^3^) can explain the functional form of noise in cognition amplifies and strengthens this argument (Zhu et al., 2022).

Even when the most probable hypothesis has been found, noise can still play a useful role. Noise allows for integrating over hypotheses that point toward the same action because the most likely hypothesis may not necessarily point to the most likely action. For example, there may be a crowd of geese running across the road, and although it may be that driving through a gap without hitting any geese with your car is most likely, integrating over all the possible errors in perception and execution means the right decision is to stop. Going further, even in situations in which the best action is already known, noise enables useful metacognitive judgments such as confidence by checking how often hypotheses that point to the best action are represented. More generally, considering all the ways that a candidate action might be wrong is necessary to not be overconfident, and it is a difficult problem not just for human cognition but for any thinking system (Moore, 2022). Indeed, it is suggestive that even advanced AI systems that internally represent probability distributions over responses tend to sample responses rather than simply choosing the most likely response because sampling in a particular way produces more realistic output (Holtzman et al., 2020).^9^

Viewed through this lens, the noise in cognitive operations is necessary, and it is unclear how much sensory or response noise is necessary to explain human behavior. Certainly, there is noise in the sensory information, and we reviewed the idea of models limited by photon noise above. Response noise could also be due to stochasticity in the environment—for example, sometimes the keys stick on the keyboard. But these sources could potentially be minor contributors, as argued by Drugowitsch et al. (2016). Moreover, variability that has been characterized as sensory or response noise in past studies could potentially be computational noise instead. Response noise can be imitated by noise earlier in the system: A common type of response noise is to assume that participants make a response according to the probability it is correct, and as we discussed above, this probability-matching behavior can be accounted for by the computational noise induced by sampling. This could help explain why cognitive load and time pressure increase probability-matching behavior (Olschewski et al., 2018; Seitz et al., 2023)—fewer samples are generated, which results in more unpredictable responding (Vul et al., 2014). What has been labeled sensory noise could be occurring after important computations have occurred—even activity in early visual areas of the brain and the retina is adaptive and could involve an inference process (Aitchison & Lengyel, 2017; Spratling, 2017). However, this is speculative, and sensory noise remains useful for explaining Weber’s law scaling of noise with stimulus magnitudes because it is not obvious why that would arise from the sampling from hypotheses. Refining models of computational noise will, in the future, allow for more accurate partitioning of the different sources of noise. In addition, investigations of computational noise may help shed new light on the representational systems used—giving new targets for which cognitive mechanisms such as similarity and rules would need to account.

## Conclusions

If this viewpoint is right, then the “noisy” nature of cognition arises from its very essence. If perception and cognition involve probabilistic inference, and that inference is carried out through sampling, then when asking a question again, different samples will, of course, generate different outcomes. Thus, we should expect human thought and behavior to be variable through and through as a reflection of the computational noise that drives the basic “engine” of cognition. Not only could one say that noise in cognition is a feature and not a bug but even that it is an essential feature, one that underpins our ability to deal with an uncertain world of such complexity that precise analysis is computationally impossible.

## Transparency

*Action Editor:* Joakim Sundh

*Editor:* Interim Editorial Panel

## Notes

1. In contrast, one aspect of stimuli that we do not consider to be external noise is the uncertainty it implies; for example, the phrases “a 50% chance of winning” and “a 100% chance of winning” have high and low implicit uncertainty, respectively. We consider implicit uncertainty to not be external noise because a fixed deterministic system would produce the same response to each presentation, whereas it would not do so in the presence of external noise.

2. Although here we focus on the visual system, there have been parallel investigations of internal noise in the auditory and haptic systems (e.g., Jepsen et al., 2008; Jones & Tan, 2012).

3. More formally, for each of the two alternatives a value, $V_{1}$ or $V_{2}$ , is assumed to be covertly computed. Each value has independent Gaussian noise added on: $V_{1}=f(S_{1})+\epsilon_{1}$ and $V_{2}=f(S_{2})+\epsilon_{2}$ , where $S_{1}$ and $S_{2}$ are the signals associated with the two alternatives and $\epsilon_{1}$ and $\epsilon_{2}$ are independent random Gaussian variables. The intended response in the decision-making task is then the alternative with the highest (noisy) value, and errors are more likely the closer that $V_{1}$ and $V_{2}$ are to one another. This formulation corresponds to a probit model of strength-dependent noise. A popular alternative is the logistic model, which corresponds to Gumbel-distributed noise on values (McFadden, 1974; Yellott, 1977). However, these two formulations do not differ much in binary-choice tasks (Luce, 1959).

4. The advantage of looking at changes in successive estimates rather than just the distribution of estimates is that it provides a more stringent test. If a participant’s estimates are constantly increasing or decreasing by a set amount on each trial with Gaussian error added on, then the distribution of changes in successive estimates will still be Gaussian, although with a nonzero mean. If participants are changing their time estimate according to a Gaussian random walk, which would be an indication of computational noise as errors are incorporated into the next estimate, then the distribution of changes between successive time interval estimates will also follow a Gaussian distribution.

5. A random walk, which could be thought of as a simple form of computational noise in which Gaussian noise is added to the immediately preceding estimate, has limited autocorrelations and would show a slope of −2 on this plot.

6. This is outside of the first lag, in which a negative autocorrelation can be explained by adjusting an estimate as the result of feedback.

7. Similarity-based models, which calculate the summed similarity of a new item to each of the response categories and respond as a function of the relative summed similarity (e.g., Medin & Schaffer, 1978; Nosofsky, 1984), can be reinterpreted as probability distributions over stimuli (Ashby & Alfonso-Reese, 1995), although responses are assumed to be drawn independently of one another.

8. A similar argument is that the noise in neurons allows it to encode probabilistic information (Echeveste & Lengyel, 2018; W. J. Ma et al., 2006).

9. Many large language models proceed in a sequential way by sampling each token (i.e., basic unit of text) conditioned on all previous tokens (Holtzman et al., 2020). Although for the first token this would therefore be response noise, the initial noise will be incorporated in later computations and so would be computational noise.
